# Medication Adherence and Contraceptive Counseling

**DOI:** 10.3390/healthcare11091304

**Published:** 2023-05-03

**Authors:** María Inmaculada de Molina-Fernandez, Laura Reyes-Martí, Miriam De la Flor-López, Maria Jesús Aguarón-García, Alba Roca-Biosca, Lourdes Rubio-Rico, Rosa Dolors Raventós Torner, Francesc Valls-Fonayet

**Affiliations:** 1 Nursing Department, Universitat Rovira i Virgili, 43002 Tarragona, Spain; inmaculada.demolina@urv.cat (M.I.d.M.-F.); laura.reyes@urv.cat (L.R.-M.); mariajesus.aguaron@urv.cat (M.J.A.-G.); alba.roca@urv.cat (A.R.-B.); lourdes.rubio@urv.cat (L.R.-R.); rosadolores.raventos@urv.cat (R.D.R.T.); 2 Medicine Department, Universitat Rovira i Virgili, 43002 Tarragona, Spain; miriamjose.delaflor@urv.cat

**Keywords:** midwifery, nursing assessment, treatment adherence and compliance, combined oral contraceptive, hormonal contraception

## Abstract

Combined oral contraceptives (COC) are a very popular form of birth control. Incorrect use and lack of adherence to treatment reduce the effectiveness of this method. Having a standard tool to identify poor-adherence profiles quickly and objectively can be helpful for midwives and potentially for COC users. The MMAS-4 adherence scale has been used in various medical fields, but there is little evidence of its potential in contraceptive consultation. This paper presents a piece of multicenter observational research based on a sample of 327 women who had attended contraceptive counselling in Spain and were COC users or had informed the midwife that they want to start to use this method. Two interviews were conducted: at the time of consultation and after one year. In our research, the MMAS-4 identified high-risk behaviors: during the 1-year follow-up period, COC users classified as poorly adherent had a significantly higher risk of missed contraceptive pills, more incidents and problems related to the method of contraception, as well as a lower degree of satisfaction with the contraceptive method. One case of unplanned pregnancy and two cases of emergency contraception were identified, all of them corresponding to poorly adherent women. The use of MMAS-4 in consultation can improve midwives’ contraceptive counselling.

## 1. Introduction

The most popular contraceptive methods are short-acting reversible contraception (SARC) methods, specifically condoms and combined oral contraceptives (COCs). In Spain, these methods, respectively, are used by 31.3% and 18.5% of women [1]. Although the condom, the most widely used method, is still the only contraceptive method that prevents sexually transmitted infections (STIs), in practice it is less effective as a contraceptive when compared to other methods, due to its inconsistent and/or incorrect use. During their first year of use, 15 out of every 100 women/year who use a condom get pregnant [2,3]. Something similar happens with COCs. Despite being theoretically more than 99% effective, their real effectiveness stands at 92%, or in other words, 8 out of 100 women/year become pregnant due to various factors that hinder adherence or due to their incorrect use [3,4].

The term adherence encompasses two concepts: dose compliance and persistence in treatment duration. The former refers to correct drug intake, and the latter to constant use over time [4]. The World Health Organization (WHO) defines treatment adherence as the extent to which a person’s behavior corresponds with the agreed recommendations from a healthcare provider [5]. In the case of COCs, adherence is understood as taking the pill daily at the same time and the series of measures observed by the user to prevent pregnancy in the event of oversight or when receiving treatments that interfere with them [6].

The lack of adherence to contraceptives in general reduces their effectiveness and results in one in three pregnancies in Spain being unplanned, of which half are interrupted [7,8]. In Spain, in 2020, 88,269 voluntary interruptions of pregnancy (VIP) were reported, of which 90.87% were performed at the request of the woman, without a clinical cause justifying them [9], with an estimated cost of some EUR 80 million [8]. The latest data from the Spanish Health Ministry show that 9.8% of women undergoing a VIP declared having used COCs [9].

During counselling, the midwife should evaluate the user’s possible adherence with respect to the contraceptive method that she will choose, since, for example, the difficulties in remembering to take a daily dose could be predictors of poor compliance in the case of COCs [4].

Quality contraceptive counseling should provide a structure that is explicitly focused on the preferences expressed by women [10]. However, professionals often warn that they have difficulties in doing so, and express the need for support interventions [10,11].

The aim of this research was to assess whether the use of a medication adherence assessment scale can help midwives when faced with a demand for contraceptive advice.

## 2. Materials and Methods

**Figure 1:**
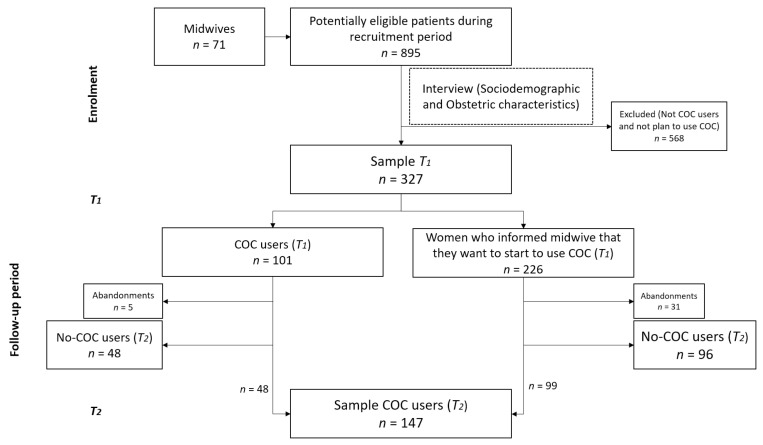
Flow diagram.

Multicenter observational research was conducted in 7 regions of Spain between July 2019 and September 2021. In total, 71 midwives from 43 Primary Healthcare Centers in Spain consecutively identified 895 women upon their attendance to the consulting room for contraceptive counselling during the enrolment period. A sample of 327 women was recruited by the professionals according to the inclusion criteria. The inclusion criteria were women who used COCs (*n* = 101) or who had informed the midwife that they would like to use COCs (*n* = 226) at the first interview (*T*_1_). The reason for establishing this filter is that the use of COCs is a highly user-dependent method that requires perfect adherence to be effective. The exclusion criteria were: (a) not understanding the Spanish language; (b) not using and not planning to use COCs; and (c) trying to get pregnant. Informed consent was obtained prior to participation in the research. All women received a follow-up during the 1-year period (*T*_2_). Of the woman recruited, 147 of them met the inclusion criteria of the second phase: being COC users (Figure 1).

The total sample size required to identify a medium effect size of w = 0.3 [12], with a power (1 − β) of 80% and an α of 0.05, was 88 cases (G*Power statistical hardware, version 3.1.9.7; χ^2^ tests—Goodness of fit tests: Contingency tables—A priori). All analyses had a larger sample than required.

Data were collected using an online questionnaire [13]. The independent variable was the result of the Spanish validated version [14] of the 1986 Morisky Medication Adherence Scale (MMAS-4), consisting of four dichotomous questions (Table 1) that evaluate the degree of pharmacotherapeutic compliance [15].

A patient was considered adherent when she answered the four items appropriately: that is, answered No/Yes/No/No. Its ease and brevity when applying it in the consultation were key factors in choosing this instrument. The demographic and obstetric characteristics of the COC users were analyzed, as well as potential risk behaviors according to the MMAS-4 classification.

Statistical analyses were conducted using SPSSv27 (IBM), using bivariate analysis (chi-square test for categorical variables and t-test for continuous variables) and a binomial logistic regression. Results were controlled for confounding variables. Significance was set at *p* < 0.05. In multiple comparisons, results were also adjusted for false discovery rate (FDR) using multiple testing software [16].

Ethics approval was obtained from the Ethics Committee of Primary Care Institute Jordi Gol (reference code: P18/208) and from the Health Research Institute (reference code CEIM: 186/2018). Prior to participating, all participants read the information sheet containing information about the study objectives and design, and information concerning the names and professional affiliations of the researchers. They then proceeded to give their informed consent by signing the relevant form. After a woman had signed the form, the professional allowed the patient access to the online questionnaire. The professional remained in the consultation room to resolve possible doubts. When the questionnaire was completed, the counseling session continued in the usual format. No negative impact on the study participants was expected.

The study was conducted in accordance with the principles of the Helsinki Declaration. Data confidentiality was protected under the Spanish Organic Law 3/2018 Protection of Personal Data.

## 3. Results

The overall sample at *T*_1_ (*n* = 327) included 101 women who were current COC users and 226 women who had informed the midwife that they wanted to use COCs. Of these patients, 60.6% were identified as poorly adherent (*n* = 198/327), including 72.3% among the current-user group (*n* = 73/101) and 55.3% among the second group (*n* = 125/226). Table 2 shows the demographic and obstetric characteristics of the sample and includes a bivariate analysis of each factor. The poorest levels of adherence were identified among married women, among those with a history of unplanned pregnancy or voluntary termination of pregnancy, among those who were mothers, among smokers, among those who were living with their partner, and among employed women. Women with poor adherence also had a higher mean age and lower blood pressure levels. No differences were found concerning weight, educational level, or place of birth.

A binomial logistic regression was performed to identify the predictive ability of all significant (*p* < 0.05) demographic and obstetric variables from Table 2 on the likelihood of poor adherence. Table 3 shows that age, diastolic blood pressure, and being a smoker remained statistically significant predictors of poor adherence. Smokers had a 2.081 times higher likelihood of poor adherence than non-smokers. Increasing age was associated with a significant increasing likelihood of poor adherence, while increasing diastolic blood pressure was associated with a significant reduction in the likelihood of poor adherence. All other variables were not defined as predictors of adherence, even though the bivariate analysis reported an association with the level of adherence.

Several factors related to the use of this contraceptive method during the one-year period from *T*_1_ to *T*_2_ were evaluated (Table 4). Among poorly adherent women, we found a 2.153 times higher probability of having forgotten to take the pill during previous 12 months (47.8% of poorly adherent women vs. 29.8% among adherent women) and of having had problems related to the use of the contraceptive method (27.8% of this group vs. 3.8% among adherent women). The degree of satisfaction with COCs (measured on a scale from 0 to 10) was significantly lower among poorly adherent women (mean = 7.92) vs. among adherent women (mean = 8.82), with a substantial effect size (Hedges’ g = 0.600, *p* < 0.001).

In all three cases (forgetting to take pills, having problems related to the use of the contraceptive method, and the degree of satisfaction), the differences remained statistically significant when controlling for being a smoker (*p* < 0.001, *p* = 0.002, and *p* = 0.006, respectively) and for age (*p* = 0.002, *p* = 0.003, and *p* = 0.004) (the significant variables of the regression model).

No statistically significant differences between adherent and poorly adherent participants were found due to any other variables studied: minor bleeding between periods (*p* = 0.274), mood changes (*p* = 0.840), decreased sexual desire (*p* = 0.071), headaches (*p* = 0.093), skin problems (*p* = 0.230), weight gain (*p* = 0.402), severe breast pain (*p* = 0.915), and nausea or vomiting (*p* = 0.307).

Finally, in the one-year follow-up period, one case of unplanned pregnancy and two cases of emergency contraception were identified, and all of these cases corresponded to poorly adherent women.

## 4. Discussion

In Spain, as in other countries, despite the numerous contraceptive options available, women continue, mostly and inconsistently, to use a reduced range of contraceptive methods whose effectiveness depends on perfect user compliance [1,17]. Research on adherence to treatment is common in other fields, such as hypertension, but it is less common in the field of contraception. Thus, the evaluation of the adherence of users who use or intend to use these methods should be checked and appraised by the professional in the consultation room [18].

The profile of adherence/poor adherence was evaluated using the MMAS-4 scale. Of women who were using COCs or intended to use them, 60.6% were identified as poorly adherent. These data coincide with those published in a 2016 review on factors related to adherence to COCs, which showed that 65–70% of users were poorly adherent, forgetting or delaying a tablet more than once a month, thus compromising the contraceptive effectiveness of the drug [4].

Similar results were published by Biset, based on a study conducted in Belgium on more than 10,000 women prescribed with isotretinoin for acne, which assessed their adherence to hormonal contraceptive methods (oral, patches, and rings); only 24.5% complied with the recommendation to use an effective contraceptive method one month before, during, and one month after treatment, despite having been informed of the teratogenic effects of the medication [19]. Similarly, other research concludes than less than 20% of women met the criteria for high adherence to oral contraceptive pills [20]. Patient education along with knowledge acquisition and perceived self-efficacy are important, not only to avoid missing a dose, but also to know how to react in these cases, especially if they are taking a teratogenic drug [20,21]. It seems clear that COCs are prescribed by clinicians during contraceptive counselling systematically without classifying the women’s user adherence status, putting a high percentage of them at risk of pregnancy [22,23,24,25,26].

In our research, once the prevalence of non-adherence was established by means of the MMAS-4, we set out to identify whether there was a sociodemographic and/or clinical profile of a greater risk of “poor adherence” among users who used or intended to use COCs. After analyzing the likelihood of poor adherence (MMAS-4) based on participants’ demographic and obstetric factors, only age, diastolic blood pressure, and smoking remained as statistically significant predictors of poor adherence. All other variables were not defined as predictors of adherence. We highlight only two of these findings, smoking and age, since diastolic blood pressure differences, though statistically significant, were within the ranges of clinical normality.

Being a smoker proved to double the likelihood of poor adherence, which should be noted, taking into account#, moreover, that the prescription of COCs to female smokers aged over 35 years would be discouraged, according to the medical eligibility criteria of the World Health Organization on the use of contraceptives [27]. Additionally, several studies warn that smoking is significantly and negatively associated with health-related quality of life, that smokers are less likely to adhere to their medication, that smokers demonstrate less adherence to protective behaviors, that smoking is linked to inadequate health literacy, and that smokers are less compliant with recommended preventive care and medication use [28,29,30,31,32].

Age is one of the most studied factors in relation to adherence, although the results of the various studies differ. In our study, increased age was associated with a significant increase in the likelihood of poor adherence. However, a study that investigated contraceptive failure in a large U.S. cohort of oral contraceptive users (52,218) showed that women aged between 20 and 24 years were three times more at risk of having an unwanted pregnancy as a result of poor adherence than women aged between 30 and 34 years [33]. In contrast, other studies showed that age was not a determining factor in adherence to COCs, since in their study, the percentages of compliance by women over 35 years of age were similar to those of younger women [34]. We identify that age (and age-related behaviors) may be a relevant factor in adherence to contraception, but this probably depends on the women’s socioeconomic and cultural context. More comparative research could provide better scientific evidence. In short, it is no easy task to identify non-adherent women based on their sociodemographic profile, as also shown by the results of a prospective cohort study carried out in Mexico in 2022 on 130 adolescents who were monitored for two years, finding no differences between the social determinants of subjects adherent and non-adherent to the contraceptive method [35]. These data match those of another study on 204 adolescents in Switzerland, which evaluated predictors for the interruption of the contraceptive method and concluded that neither age, nationality, smoking, nor occupation influenced adherence [36].

We also found that increased diastolic blood pressure was associated with a significant reduction in the likelihood of poor adherence. This could be related to better self-care behavior, health management, and better health literacy. However, it is a specific line of research that should be studied in the future.

Finally, our study sample was followed up after one year. After the analysis, 52% of female users of COCs were found to have abandoned the method within a year of starting it. These results coincide with those of Lete, which show that approximately half of women in Spain abandon COCs within 12 months of starting treatment due to the onset of side effects and/or lack of adherence [37]. It was also noted that women classified by the MMAS-4 as “non-compliant” were more likely to have forgotten to take COCs and had experienced problems related to the use of the contraceptive method, also presenting a significantly lower degree of satisfaction with the method than that shown by adherent women. COCs are a contraceptive method whose effectiveness requires strict adherence to the administration guidelines [38]. The use of a simple scale in the consultation room that helps identify women requesting to use COCs who may be exposed to the risk of an unwanted pregnancy could be the key.

New and emerging approaches are being reviewed to support improved adherence to contraceptive methods. In this regard, Huber et al. conducted a clinical trial to evaluate the usefulness of monitoring journals for COC users, establishing that the overall outcomes were not as satisfactory as they were expecting [39]. Another involved assessing the effectiveness of sending text messages as a reminder, based on an experimental study, which concluded that the mean number of forgotten pills per cycle did not differ significantly between the experimental and control groups [40]. In a 2019 Cochrane review of the effectiveness of strategies to improve compliance with and the continuation of short-acting reversible contraception (SARC), it was established that it was not clear whether reminders improved the continuation of hormonal contraceptive methods compared to usual healthcare [41]. This was corroborated in a study by Nguyen in 2021, who found no differences in the number of forgotten pills when comparing the use of technological reminder systems with traditional ones [42]. These results suggest that there are women who comply excellently and, as such, are candidates for COCs, and there are others who, if they are not excellent compliers, will adhere poorly to COCs, even if reminder systems are implemented.

In this scenario, when poor adherence is identified, the option of using long-acting reversible contraception (LARC) methods, which include the implant, the hormonal IUD, and the copper IUD, is raised, since their effectiveness is close to 99% despite user non-compliance or incorrect use [43]. However, the results of a meta-analysis on contraceptive effectiveness showed that despite the advantages of LARC methods, young women continued mostly to use SARC methods [44].

In Spain, according to recent data, 64.3% of women attended a clinic (family doctor, gynecologist, midwife, or other) to receive advice concerning the most suitable method of contraception in their case. Among women who have chosen the contraceptive pill or the IUD, the clinician is considered the prescriber with the greatest final influence [1].

Dehlendorf, the author of an interesting review on how contraceptive counselling should be performed, insists on the need for clinicians to establish a close, trusting relationship with the user, focused on finding out about her personal situation and lifestyle, as well as her values and preferences in this regard [45]. Aspects such as having little information about the method, a lack of knowledge regarding forgetfulness, not starting it immediately, a high cost, lack of support from the partner, not participating in the choice of the method, and difficulties in agreeing to use it are considered predictors of poor compliance [3,4,18].

It is therefore proposed that, given the uncertainty of factors that clearly discriminate the lack of user adherence, the routine application of the MMAS-4 (already used in other areas of primary care [46]) in contraceptive counselling consultations, would enable users to be informed of this condition, and, on this basis, advised on the most effective and safest methods for her.

## 5. Conclusions

One of the contraceptive methods most used by Spanish women is the combined oral contraceptive (COC). The effectiveness of COCs requires strict adherence to treatment by the user. Identifying profiles of users who might not meet this requirement is a fundamental task of midwives’ contraceptive counselling.

Our results reported that 72.3% of COC users and 55.1% of women who had planned to start to use COC were poorly adherent, based on the MMAS-4 scale. Older age, being a smoker, and lower diastolic blood pressure were identified as predictors of low adherence. However, the condition of poor adherence was generalized among the other sociodemographic and clinical profiles studied, according to the regression model.

The MMAS-4 adherence scale correctly identified risk profiles of poorly adherent women. At a follow-up interview after one year, women using COC and classified as poorly adherent reported more forgetfulness in taking birth control pills, more problems related to the contraceptive method, and a lower degree of satisfaction with the contraceptive method. Two women using COC reported unplanned pregnancies and another resorted to emergency contraception. All of these women were identified as poorly adherent by the MMAS-4 scale.

The benefits of using a poor-adherence screening tool, such as the MMAS-4, including that is easy to use and allows for the correct identification of risk behaviors, should be considered.

### Limitations

There is no gold-standard test that can be used for any patient. Despite the relevance of adherence in oral contraception, there is no specific adherence scale for COC. The MMAS-8 scale [47] has better psychometric properties, but the MMAS-4 is also a highly validated scale. In addition, this four-item scale can more quickly assess a patient’s medication-taking behavior. Potential sources of bias were identified. For example, subjective bias could appear, in the sense that when using the MMAS-4 scale, the subjects might have answered what they believed the researcher/professional considered appropriate. On the other hand, a recall bias could also appear, for example, if subjects who had experienced incidents while taking COC better remembered a lack of adherence than subjects who hadn’t experienced incidents. Due to the subjective nature of these answers, further research could incorporate an objective measure of adherence (such as the Medication Possession Ratio).

## Figures and Tables

**Table 1:** Items for the Morisky Medication Adherence Scale (MMAS–4).

Item	Answer
1. Do you ever forget to take your medicine?	Yes/No
2. Are you careless at times about taking your medicine?	Yes/No
3. When you feel better, do you sometimes stop taking your medicine?	Yes/No
4. Sometimes if you feel worse when you take your medicine, do you stop taking it?	Yes/No

**Table 2:** Demographic and obstetric characteristics of the sample, organized by MMAS-4 results of adherence and poor adherence *n* = 327 at *T*_1_.

Categorical Factors	Total	Poorly Adherent	Adherent	*p*-Value
	*n* = 327	*n* = 198	*n* = 129	
	(% of Total)	(% of Row)	(% of Row)	
Marital status				0.010
Married	54 (16.5%)	41 (75.9%)	13 (24.1%)	
Single or other	270 (82.6%)	154 (57.0%)	116 (43.0%)	
Has children				0.003
Yes	99 (30.3%)	72 (72.7%)	27 (27.3%)	
No	228 (69.7%)	126 (55.3%)	102 (44.7%)	
Living with a partner				0.002
Yes	149 (45.6%)	104 (69.8%)	45 (30.2%)	
No	178 (54.4%)	94 (52.8%)	84 (47.2%)	
Activity situation				0.002
Employed	179 (54.7%)	122 (68.2%)	57 (31.8%)	
Non-employed	146 (44.6%)	75 (51.4%)	71 (48.6%)	
History of unplanned pregnancy				0.037
Yes	64 (19.6%)	46 (71.9%)	18 (28.1%)	
No	262 (80.1%)	151 (57.6%)	111 (42.4%)	
Voluntary pregnancy interruptions				0.045
Yes	49 (15.0%)	36 (73.5%)	13 (26.5%)	
No	278 (85.0%)	162 (58.3%)	116 (41.7%)	
Smoker				0.027
Yes	74 (22.6%)	53 (71.6%)	21 (28.4%)	
No	253 (77.4%)	145 (57.3%)	108 (42.7%)	
Level of education				0.999
Lower Secondary	112 (34.3%)	68 (60.7%)	44 (39.3%)	
Upper Secondary	104 (31.8%)	62 (60.6%)	41 (39.4%)	
University	111 (33.9%)	67 (60.4%)	44 (39.6%)	
Place of birth				0.120
Spain	222 (67.9%)	128 (57.7%)	94 (42.3%)	
Abroad	105 (32.1%)	70 (66.7%)	35 (33.3%)	
**Continuous Factors**	**Mean ± S.E. of Mean**	**Mean ± S.E. of Mean**	**Mean ± S.E. of Mean**	***p***
Age	26.13 ± 0.40	27.45 ± 0.51	24.11 ± 0.58	<0.001
Weight	63.04 ± 0.69	63.79 ± 0.85	61.88 ± 1.17	0.177
Systolic blood pressure	113.2 ± 0.62	112.11 ± 0.78	114.9 ± 0.99	0.027
Diastolic blood pressure	69.99 ± 0.46	69.03 ± 0.58	70.5 ± 0.73	0.008

All significant *p*-values were significant using multiple comparison correction (Benjamini–Hochberg adjusted *p*-value). S.E., standard error of mean. No imputation was made for missing values: 3/327 for marital status; 2/327 for activity situation; 1/327 for history of unplanned pregnancy; 2/327 for systolic blood pressure, and 3/327 for diastolic blood pressure. Source: authors’ own, based on data from the research.

**Table 3:** Binomial logistic regression predicting likelihood of poor adherence (MMAS-4) based on demographic and obstetric factors. *n* = 314 at *T*_1_.

	B	Standard Error	Wald	df	*p*	OR	95% CI for OR	
							Lower	Upper
**Categorical Predictors**								
Marital status								
Married (Ref.)								
Single or other	−0.879	0.486	3.271	1	0.070	0.415	0.160	1.076
Has children								
No (Ref.)								
Yes	0.084	0.424	0.040	1	0.842	1.088	0.474	2.496
Living with a partner								
No (Ref.)								
Yes	0.057	0.320	0.032	1	0.859	1.059	0.565	1.982
Activity situation								
Employed (Ref.)								
Non-employed	−0.094	0.293	0.104	1	0.748	0.910	0.512	1.617
History of unplanned pregnancy								
No (Ref.)								
Yes	0.020	0.691	0.001	1	0.977	1.020	0.263	3.951
Voluntary Pregnancy Interruptions								
No (Ref.)								
Yes	0.375	0.737	0.259	1	0.611	1.455	0.343	6.164
Smoker								
No (Ref.)								
Yes	0.733	0.320	5.243	1	0.022	2.081	1.111	3.898
**Continuous Predictors**								
Age	0.075	0.027	7.511	1	0.006	1.078	1.022	1.137
Systolic blood pressure	−0.017	0.014	1.405	1	0.236	0.983	0.957	1.011
Diastolic blood pressure	−0.043	0.019	5.075	1	0.024	0.958	0.923	0.994
Constant	4.016	1.567	6.571	1	0.010	55.478		

The logistic regression model was statistically significant, χ^2^ = 49.931(10), *p* < 0.001. The model explained 20.0% (Nagelkerke R^2^) of the variance and correctly classified 67.2% of cases. Sensitivity (a correct classification of poorly adherent women) was 79.8%, specificity was 47.1%, the positive predictive value was 71.3%, and the negative predictive value was 59.4%. All significant *p*-values were significant using multiple comparison correction (Benjamini–Hochberg adjusted *p*-value). Of the sample *n* = 327, 13 cases were not included due to missing values (*n* = 9) and after an analysis of outliers (*n* = 4). Source: authors’ own, based on data from the research.

**Table 4:** Risk elements in the use of COCs at *T*_2_, during the previous year (from *T*_1_ to *T*_2_), organized according to MMAS-4 results of adherence and poor adherence. *n* = 147 at *T*_2_.

Categorical Factors	Total (% of Total COC Users, *n* = 147)	Poorly Adherent (% of Poorly Adherent, *n* = 90)	Adherent (% of Adherent, *n* = 57)	OR (95% CI)	*p*-Value	Effect Size (φ)
Forgetting to take pills	60	43	17	2.153	0.031	0.178
	(40.8%)	(47.8%)	(29.8%)	(1.067–4.345)		
Some incidents or problems related to contraceptive method (COCs)	27	25	2	9.808	<0.001	0.296
	(18.9%)	(27.8%)	(3.8%)	(2.219–43.353)		
Bleeding between periods	41	28	13	1.529	0.274	-
	(27.9%)	(31.1%)	(22.8%)	(0.713–3.278)		
Mood changes	45	27	18	.929	0.840	-
	(30.6%)	(30.0%)	(31.6%)	(0.453–1.903)		
Decreased sexual desire	29	22	7	2.311	0.071	-
	(19.7%)	(22.4%)	(12.3%)	(0.916–5.831)		
Headaches	34	25	9	2.051	0.093	-
	(23.1%)	(27.8%)	(15.8%)	(0.878–4.791)		
Skin problems	22	16	6	1.838	0.230	-
	(15.0%)	(17.8%)	(10.5%)	(0.674–5.015)		
Weight gain	31	21	10	1.430	0.402	-
	(21.1%)	(23.3%)	(17.5%)	(0.618–3.311)		
Severe breast pain	42	26	16	1.041	0.915	-
	(28.6%)	(28.9%)	(28.1%)	(0.499–2.173)		
Nausea or vomiting	18	13	5	1.756	0.307	-
	(12.2%)	(14.4%)	(8.8%)	(0.590–5.221)		
Unplanned pregnancy	1	1	0	-	-	-
	(0.7%)	(1.1%)	(0%)			
Has needed emergency contraception	2	2	0	-	-	-
	(1.4%)	(2.2%)	(0%)			
**Continuous Factors**	**Mean ± SD**	**Mean ± SD**	**Mean ± SD**	***p*** **-value**		**Effect size (Hedges’ g)**
Degree of satisfaction with contraceptive method	8.27 ± 1.56	7.92 ± 1.750	8.82 ± 0.984	<0.001		0.600

All significant *p*-values were significant using multiple comparison correction (Benjamini–Hochberg adjusted *p*-value). Source: authors’ own, based on data from the research.

## Data Availability

The data from the current study are available from the corresponding author upon reasonable request.
